# Constraining the Pluripotent Fate of Human Embryonic Stem Cells for Tissue Engineering and Cell Therapy – The Turning Point of Cell-Based Regenerative Medicine

**DOI:** 10.9734/BBJ/2013/4309

**Published:** 2013-07-13

**Authors:** Xuejun H. Parsons

**Affiliations:** 1 San Diego Regenerative Medicine Institute, San Diego, CA 92109, USA.; 2 Xcelthera, San Diego, CA 92109, USA.

**Keywords:** Human embryonic stem cell, stem cell, pluripotent, tissue engineering, cell therapy, regenerative medicine, neurological disease, heart disease

## Abstract

To date, the lack of a clinically-suitable source of engraftable human stem/progenitor cells with adequate neurogenic potential has been the major setback in developing safe and effective cell-based therapies for regenerating the damaged or lost CNS structure and circuitry in a wide range of neurological disorders. Similarly, the lack of a clinically-suitable human cardiomyocyte source with adequate myocardium regenerative potential has been the major setback in regenerating the damaged human heart. Given the limited capacity of the CNS and heart for self-repair, there is a large unmet healthcare need to develop stem cell therapies to provide optimal regeneration and reconstruction treatment options to restore normal tissues and function. Derivation of human embryonic stem cells (hESCs) provides a powerful *in vitro* model system to investigate molecular controls in human embryogenesis as well as an unlimited source to generate the diversity of human somatic cell types for regenerative medicine. However, realizing the developmental and therapeutic potential of hESC derivatives has been hindered by the inefficiency and instability of generating clinically-relevant functional cells from pluripotent cells through conventional uncontrollable and incomplete multi-lineage differentiation. Recent advances and breakthroughs in hESC research have overcome some major obstacles in bringing hESC therapy derivatives towards clinical applications, including establishing defined culture systems for *de novo* derivation and maintenance of clinical-grade pluripotent hESCs and lineage-specific differentiation of pluripotent hESCs by small molecule induction. Retinoic acid was identified as sufficient to induce the specification of neuroectoderm direct from the pluripotent state of hESCs and trigger a cascade of neuronal lineage-specific progression to human neuronal progenitors and neurons of the developing CNS in high efficiency, purity, and neuronal lineage specificity by promoting nuclear translocation of the neuronal specific transcription factor Nurr-1. Similarly, nicotinamide was rendered sufficient to induce the specification of cardiomesoderm direct from the pluripotent state of hESCs by promoting the expression of the earliest cardiac-specific transcription factor Csx/Nkx2.5 and triggering progression to cardiac precursors and beating cardiomyocytes with high efficiency. This technology breakthrough enables direct conversion of pluripotent hESCs into a large supply of high purity neuronal cells or heart muscle cells with adequate capacity to regenerate CNS neurons and contractile heart muscles for developing safe and effective stem cell therapies. Transforming pluripotent hESCs into fate-restricted therapy derivatives dramatically increases the clinical efficacy of graft-dependent repair and safety of hESC-derived cellular products. Such milestone advances and medical innovations in hESC research allow generation of a large supply of clinical-grade hESC therapy derivatives targeting for major health problems, bringing cell-based regenerative medicine to a turning point.

## 1. INTRODUCTION

Pluripotent human embryonic stem cells (hESCs) have both the unconstrained capacity for long-term stable undifferentiated growth in culture and the intrinsic potential for differentiation into all somatic cell types in the human body, holding tremendous potential for restoring human tissue and organ function [1–3]. Derivation of hESCs, essentially the *in vitro* representation of the pluripotent inner cell mass (ICM) or epiblast of the human blastocyst, provides not only a powerful *in vitro* model system for understanding human embryonic development, but also an unlimited source for *in vitro* derivation of a large supply of disease-targeted human somatic cells for tissue engineering and cell therapy. There is a large unmet healthcare need to develop hESC-based therapeutic solutions to provide optimal regeneration and reconstruction treatment options for normal tissue and function restoration in many devastating and life-threatening diseases and injuries. However, realizing the developmental and therapeutic potential of hESC derivatives has been hindered by conventional approaches for generating functional cells from pluripotent cells through uncontrollable, incomplete, and inefficient multi-lineage differentiation [2,3]. Conventional approaches rely on multi-lineage inclination of pluripotent cells through spontaneous germ layer differentiation, which yields embryoid body (EB) consisting of a mixed population of cell types that may reside in three embryonic germ layers and results in inefficient, incomplete, and uncontrollable differentiation that is often followed by phenotypic heterogeneity and instability, hence, a high risk of tumorigenicity [1–9]. Growing evidences indicate that incomplete lineage specification of pluripotent cells via multi-lineage differentiation often resulted in poor performance of such stem cell derivatives and/or tissue-engineering constructs following transplantation [2,3,10]. In order to generate a large supply of uniform functional cells for tissue engineering and cell therapy, how to channel the wide differentiation potential of pluripotent hESCs efficiently and predictably to a desired lineage has been a major challenge for clinical translation. In addition, most currently available hESC lines were derived and maintained on animal feeder cells and proteins, therefore, such hESCs have been contaminated with animal biologics and unsuitable for clinical application [2,3,11–13]. Without a practical strategy to convert pluripotent cells direct into a specific lineage, previous studies and profiling of hESC differentiating multi-lineage aggregates have compromised their implications to molecular controls in human embryonic development. Developing novel strategies for well-controlled efficiently directing pluripotent hESCs exclusively and uniformly towards clinically-relevant cell types in a lineage-specific manner is not only crucial for unveiling the molecular and cellular cues that direct human embryogenesis, but also vital to harnessing the power of hESC biology for tissue engineering and cell-based therapies.

To date, the lack of a clinically-suitable source of engraftable human stem/progenitor cells with adequate neurogenic potential has been the major setback in developing safe and effective cell-based therapies for regenerating the damaged or lost central nervous system (CNS) structure and circuitry in a wide range of neurological disorders. Similarly, the lack of a clinically-suitable human cardiomyocyte source with adequate myocardium regenerative potential has been the major setback in regenerating the damaged human heart. Given the limited capacity of the CNS and heart for self-repair, transplantation of hESC neuronal and heart cell therapy derivatives holds enormous potential in cell replacement therapy. Clinical applications of hESC therapy derivatives provide the right alternative for many incurable diseases and major health problems that the conventional mode of drugs and treatments cannot, such as heart disease and failure, diabetes, Parkinson’s diseases, ALS, Alzheimer disease, stroke, brain and spinal cord injuries. Each single one of those world-wide major health problems would cost the health care system more than $10 billion annually.

**Fig. 1:**
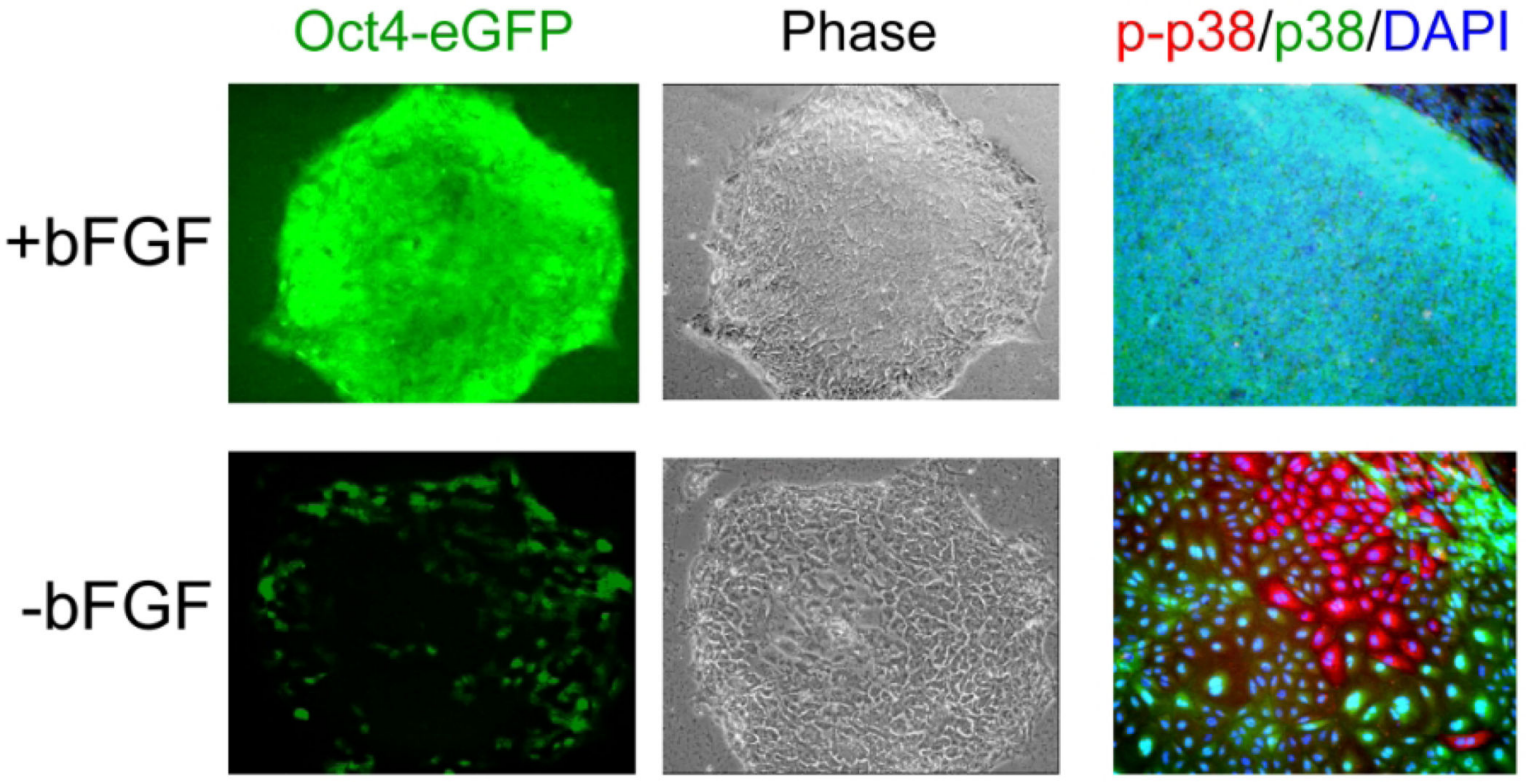
bFGF is one of the minimal essential defined elements for the maintenance of undifferentiated hESCs Representative Oct-4-driven eGFP+ hESC colonies are shown in the absence of bFGF (−bFGF) and in the presence of 20 ng/ml bFGF (+bFGF). In media lacking bFGF, hESC colonies maintained on laminin/collagen down-regulate Oct-4 expression (green) and have a completely differentiated morphology (Phase). An unphosphorylated inactive form of p38 (green cells) was observed in undifferentiated hESCs maintained in the defined media containing 20 ng/ml bFGF [+bFGF]. Although, in the absence of bFGF, the unphosphorylated form of p38 remained present in most of the large cells inside the differentiated hESC colony, a subpopulation (~5%) of the large differentiated cells displayed high level of p38 phosphorylation [p-p38, red cells] [−bFGF], suggesting that the p38 MAPK signaling was activated and might be involved in differentiation of those cells. All cells are indicated by DAPI staining of their nuclei (blue).

**Fig. 2:**
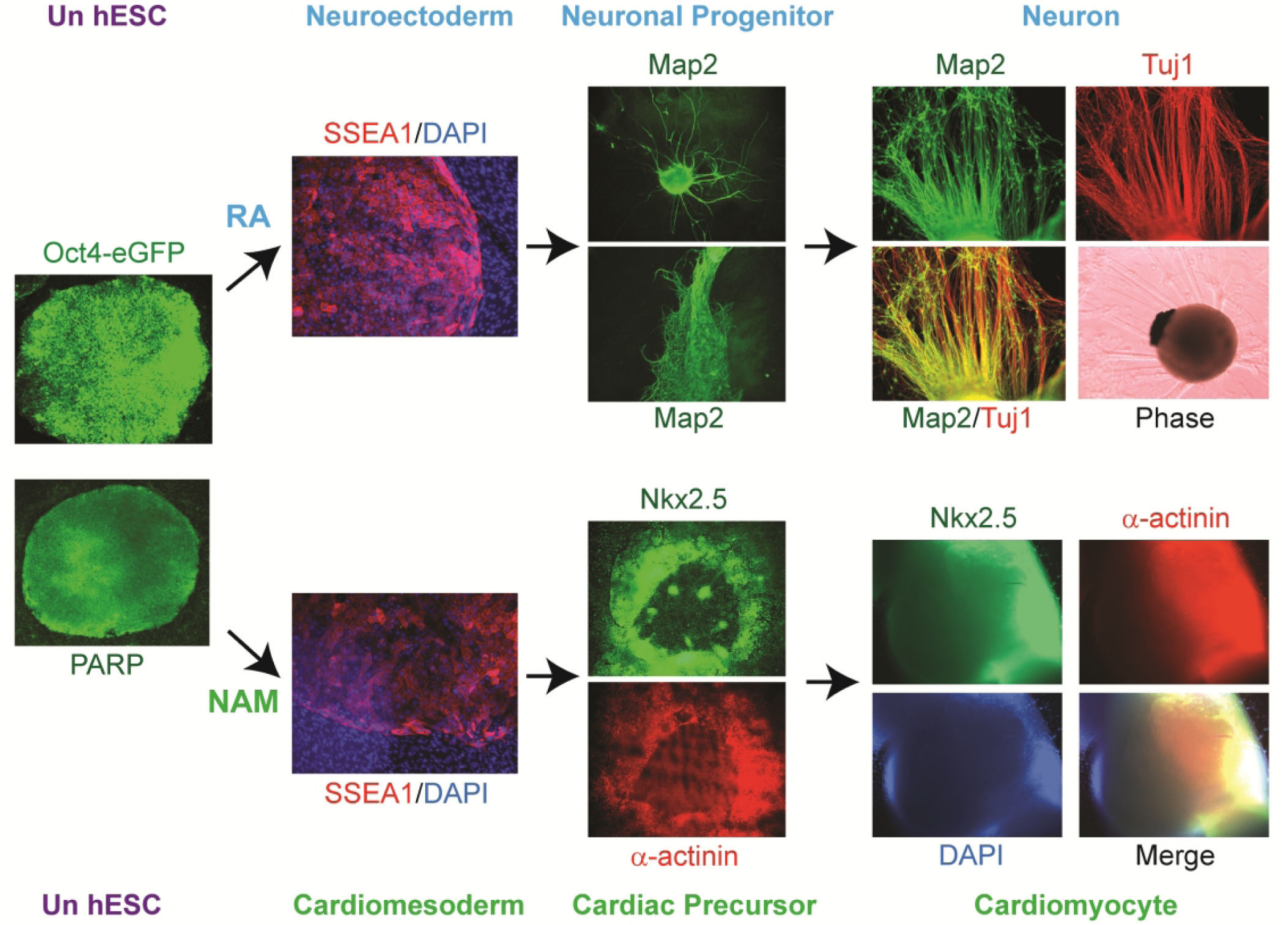
Evolution of specific somatic cell type direct from pluripotent hESCs by lineage-specific induction with signal molecules Formulation of minimal essential defined conditions for hESCs renders pluripotent hESCs be uniformly converted into a specific neural or cardiac lineage by small signal molecule induction and progress exclusively down either the neuronal lineage with retinoic acid (RA) induction or the cardiomyocyte lineage with nicotinamide (NAM) induction [3, 12, 15, 17]. Under defined culture, RA induces the specification of neuroectoderm, indicating by SSEA-1 (red) expression, direct from the pluripotent state of undifferentiated hESCs (Un hESC), expressing Oct-4 (Oct-4-driven eGFP+ hESC colonies, green) and PARP-1 (green), and trigger a cascade of neuronal lineage-specific progression to human neuronal progenitors (hESC-I hNuP), indicated by the beginning of Map-2 (green) expression, and neurons (hESC-I hNu), indicated by Map-2 (green) and Tuj1 (beta-III-tubulin, red) expression, of the developing CNS in high efficiency, purity, and neuronal lineage specificity. Similarly, under the defined culture, NAM induces the specification of cardiomesoderm, indicating by SSEA-1 (red) expression, direct from the pluripotent state of hESCs by promoting the expression of the earliest cardiac-specific transcription factor Csx/Nkx2.5 and triggering progression to cardiac precursors, indicated by Nkx2.5 (green) and alpha-actinin (red) expression, and beating cardiomyocytes, indicated by Nkx2.5 (green) and alpha-actinin (red) expression, with high efficiency. All cells are indicated by DAPI staining of their nuclei (blue).

**Fig. 3:**
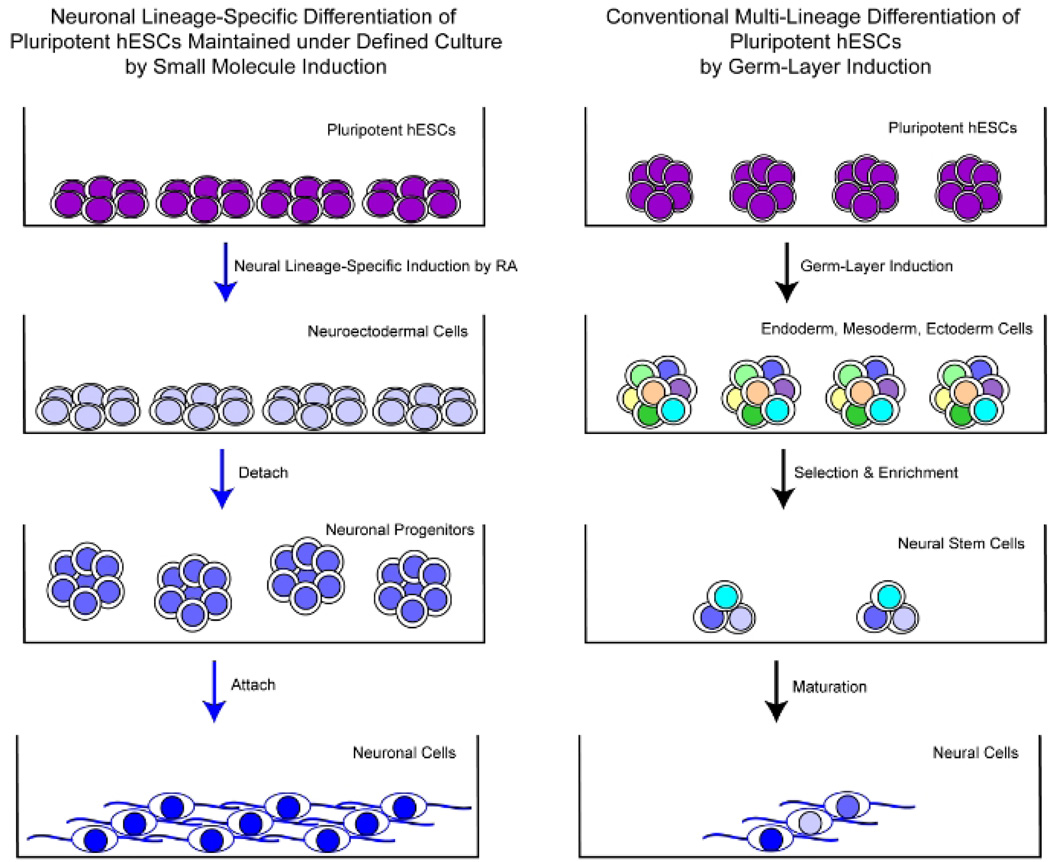
Schematic comparison of well-controlled efficient neuronal lineage-specific differentiation of pluripotent hESCs maintained under defined culture exclusively to a neuronal fate by small signal molecule induction [3,14,16–21] versus conventional neural differentiation approach using multi-lineage inclination of pluripotent cells through spontaneous germ layer induction [22–25,79–90]

**Fig. 4:**
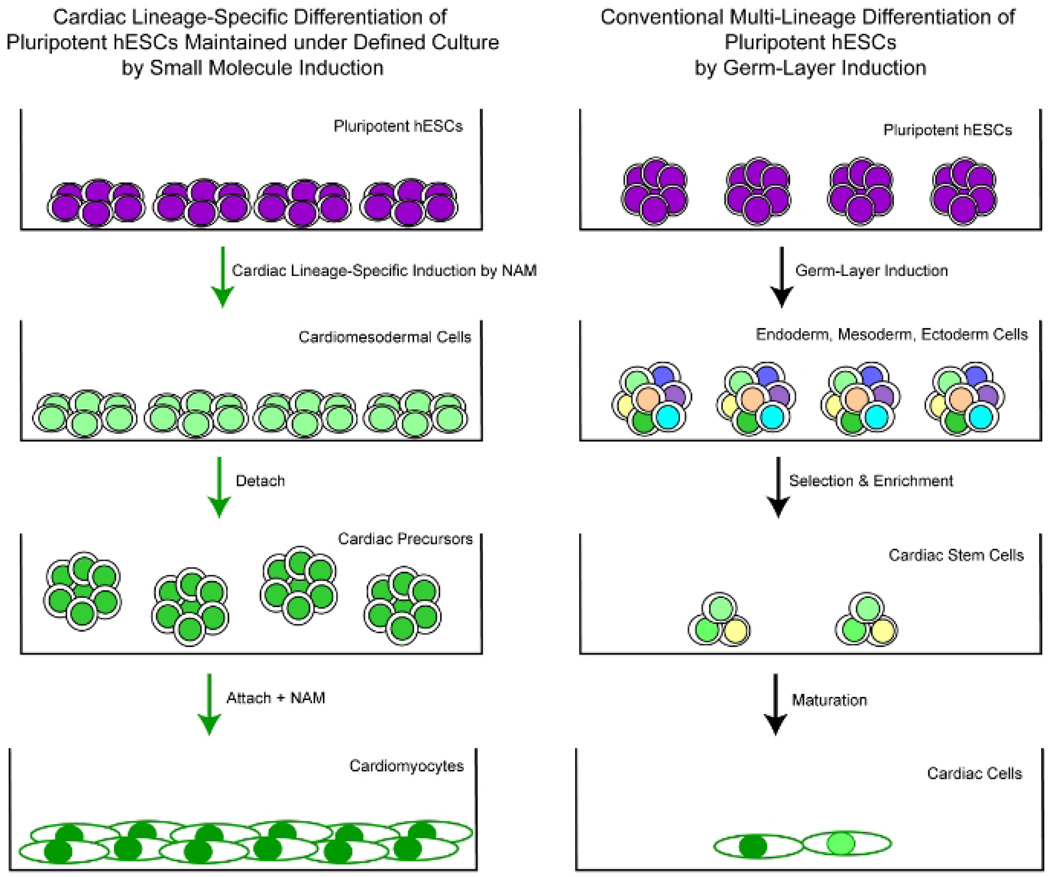
Schematic comparison of well-controlled efficient cardiac lineage-specific differentiation of pluripotent hESCs maintained under the defined culture exclusively to a cardiomyocyte fate by small signal molecule induction [3,12–16,21] versus conventional cardiac differentiation approach using multi-lineage inclination of pluripotent cells through spontaneous germ layer induction [26,27,115–125]

Recent advances and breakthroughs in hESC research have overcome some major obstacles in bringing hESC therapy derivatives towards clinical applications, including establishing human stem cell technology platforms for defined culture systems for derivation and maintenance of clinical-grade pluripotent hESCs and lineage-specific differentiation of pluripotent hESCs by small signal molecule induction for direct conversion of pluripotent hESCs into a large supply of high purity neuronal cells or heart muscle cells with adequate capacity to regenerate neurons and contractile heart muscles for developing safe and effective stem cell therapies [3,12–21]. bFGF, insulin, ascorbic acid, laminin, and activin-A were identified as the minimal essential elements for sustaining pluripotence of hESCs in a defined culture system (Fig. 1), serving as a platform for *de novo* derivation of therapeutically-suitable pluripotent hESCs that can be directly converted into large supplies of safely engraftable neuronal or cardiac lineage-committed progenies for neural or cardiac repair in the clinical setting [3,12–21]. Formulation of minimal essential defined conditions for hESCs renders pluripotent hESCs be uniformly converted into a specific neural or cardiac lineage by small signal molecule induction [3,12–21] (Figs. 2–4). Retinoic acid (RA) was identified as sufficient to induce the specification of neuroectoderm direct from the pluripotent state of hESCs and trigger a cascade of neuronal lineage-specific progression to human neuronal progenitors (hESC-I hNuP) and neurons (hESC-I hNu) of the developing CNS in high efficiency, purity, and neuronal lineage specificity by promoting nuclear translocation of the neuronal specific transcription factor Nurr-1 [3,14,16–21] (Figs. 2, 3). Similarly, nicotinamide (NAM) was identified sufficient to induce the specification of cardiomesoderm direct from the pluripotent state of hESCs by promoting the expression of the earliest cardiac-specific transcription factor Csx/Nkx2.5 and triggering progression to cardiac precursors and beating cardiomyocytes with high efficiency [3,12–16] (Figs. 2, 4). Such milestone advances and medical innovations in hESC research enable generation of a large supply of high purity clinical-grade hESC neuronal and heart cell therapy products for treating neurological and heart diseases and injuries. Currently, these hESC neuronal and cardiomyocyte therapy derivatives are the only available human cell sources with adequate capacity to regenerate neurons and contractile heart muscles, vital for CNS and heart repair in the clinical setting. The availability of human stem/progenitor/precursor cells in high purity and large quantity with adequate neurogenic or cardiogenic potential will greatly facilitate developing safe and effective cell-based regeneration and replacement therapies against CNS and heart disorders. Clinical translation of milestone advances and medical innovations in hESC research provides the only hope to many devastating and life-threatening diseases and injuries. Transforming pluripotent hESCs into fate-restricted therapy derivatives dramatically increases the clinical efficacy of graft-dependent repair and safety of hESC-derived cellular products, bringing cell-based regenerative medicine to a turning point.

## 2. Defined Platform for Well-Controlled Derivation, Maintenance, and Differentiation of Clinical-grade Pluripotent hESCs

The hESCs have the capacity for long-term undifferentiated growth in culture and the theoretical potential for differentiation into all somatic cell types [1]. Pluripotent hESCs have been shown to generate teratomas *in vivo* as well as differentiate into many different lineages and cell types *in vitro*, including neural precursors, glia, neurons, cardiomyocytes, hematopoietic precursors, endodermal and endocrine cells, and skeletal myoblasts by allowing multi-lineage differentiation through aggregate formation in suspension or extended culture [22–31]. However, only a small fraction of those cells progresses to display targeted differentiation characteristics. Although procedures such as immunoselection for specific surface antigens, treating floating aggregates (embryoid body [EB]) with inducing molecules, coculturing with mouse stromal cells, or manipulating cell density or serum concentration appeared to enrich the populations of desired cell types, none has been able to produce a large population of uniform uncontaminated functional progenies from hESCs for therapeutic application [2]. In addition, the simultaneous emergence of substantial widely divergent uncharacterized cell types that may reside in all three embryonic germ-layers in the aggregates makes directing hESC differentiation en a particular route unpredictable and unreliable, compromising the therapeutic potential of hESCs [2]. Without a through understanding of the molecular and cellular cues that direct hESC differentiation programs, controlled differentiation of hESCs effectively into functional lineages has proven to be one of the daunting challenges for fulfilling the therapeutic promise of hESCs.

Maintaining undifferentiated hESCs in a defined biologics-free culture system that allows faithful expansion and controllable direct differentiation is one of the keys to their therapeutic utility and potential, which requires a better understanding of the minimal essential components necessary for sustaining the pluripotent state and well-being of undifferentiated hESCs [2,3]. The hESC lines initially were derived and maintained in co-culture with growth-arrested mouse embryonic fibroblasts (MEFs) [1]. Using this mouse-support system may compromise the therapeutic potential of these hESCs because of the risk of transmitting xenopathogens, altering genetic background, and promoting the expression of immunogenic proteins [3,32]. In addition, the need for foreign biologics for derivation, maintenance, and differentiation of hESCs may make direct use of such cells and their derivatives in patients problematic [3].

To avoid those shortcomings, several human feeder, feeder-free, recombinant laminin, and artificially-formulated defined culture systems have been developed for derivation and maintenance of hESCs, though the elements for sustaining prolonged stable undifferentiated growth in those culture systems remain unsolved [33–39]. These exogenous feeder cells and molecules help maintain the long-term stable growth of undifferentiated hESCs while mask their ability to respond to differentiation inducing signals and molecules. Without an understanding of the essential developmental components for sustaining hESC pluripotence and self-renewal, such hESC lines are at risk for becoming unhealthy and unstable after prolonged culturing under artificially-formulated chemically-defined conditions [3]. Achieving effective differentiation of hESCs into a specific lineage, first and at least, requires a better understanding of the elements necessary and sufficient for sustaining the pluripotence of hESCs, a platform from which controlled differentiation can then directly proceed from the earliest developmental stage [3].

To overcome some of the major obstacles in basic biology and therapeutic application of hESCs, our recent studies have resolved the elements of a defined culture system necessary and sufficient for sustaining the epiblast pluripotence of hESCs, serving as a platform for *de novo* derivation of animal-free therapeutically-suitable hESCs and well-controlled efficient specification of such pluripotent cells exclusively and uniformly towards a particular lineage by small molecule induction [3,12,14] (Fig. 1). bFGF (at an optimal concentration of 20 ng/ml), insulin (20 µg/ml), ascorbic acid (50 µg/ml), laminin, and activin-A (50 ng/ml) were identified as the minimal essential elements for sustaining pluripotence and self-renewal of clonal hESCs in a defined culture system, serving as a platform for *de novo* derivation of therapeutically-suitable pluripotent hESCs that can be directly induced by small molecules into large supplies of safely engraftable neuronal or cardiac lineage-committed progenies across the spectrum of developmental stages with adequate CNS or myocardial regenerative potential for neural or cardiovascular repair in the clinical setting [3,12,14]. Establishing defined platform for the long-tern stable maintenance of pluripotent hESCs has overcome some of the major obstacles in translational biology. Good manufacturing practice (GMP) quality, defined by both the European Medicine Agency (EMA) and the Food and Drug Administration (FDA), is a requirement for clinical-grade cells, offering optimal defined quality and safety in cell transplantation [11]. Resolving minimal essential requirements for the maintenance of pluripotent hESCs allows all poorly-characterized and unspecified biological additives, components, and substrates in the culture system, including those derived from animals, to be removed, substituted, or optimized with defined human alternatives for optimal production GMP-quality xeno-free hESC lines and their therapy derivatives [3,12].

Maintaining pluripotent hESCs in a defined culture enables the spontaneous unfolding of early embryogenic processes *in vitro* that emulate the *in vivo* maintenance of the pluripotent epiblast [3,12]. In early embryogenesis, the epiblast is composed of more progressed pluripotent cells developed from the ICM, serving as the most immediate precursors of the early somatic lineages [40–44]. We found that such defined conditions derived their efficacy from enabling the spontaneous unfolding of inherent early embryogenesis processes *in vitro* that emulated the maintenance of the pluripotent epiblast developed from the ICM *in vivo*. Therefore, such defined culture system not only rendered specification of clinically-relevant early lineages directly from the pluripotent state without an intervening multi-lineage germ-layer stage, but also allowed identify the signaling molecules necessary and sufficient for inducing the cascade of organogenesis in a process that might emulate the human embryonic development [3,12].

The hESCs are not only pluripotent, but also incredibly stable and positive, as evident by that only the positive active chromatin remodeling factors, but not the negative repressive chromatin remodeling factors, can be found in the pluripotent epigenome of hESCs [14,18, 20,21]. In the last few years, pluripotency-inducing factors, most of which are known oncogenes, have been used to reprogram somatic cells to induced pluripotent stem cells (iPS cells) [44–47]. However, the extremely low efficiencies (< 0.1%) of the iPS cell technique diminish its clinical implication. The scientific definition and proof for stem cells are that they have the intrinsic ability of both self-renewal and differentiation. Pluripotent hESCs can maintain prolonged normal stable growth or self-renewal in non-hostile growth environments containing the essential developmental components that sustain hESC pluripotence and self-renewal [3,12]. However, so far, there is no evidence that pluripotent cells derived from sources harboring adult nuclei by somatic cell nuclear transfer or transcription-factor-based reprogramming or small-molecule-based reprogramming, such as iPS cells or ESC derived from cloned embryos, can maintain prolonged normal stable growth or self-renewal [44–50]. In addition, some techniques that those reports used for their analysis of pluripotent sticky cells, such as FACS for sorting non-sticky adult cells or western blot analysis for detecting weakly expressed molecules that cannot be detected by immunocytochemical analysis, would give false positive to a heterogeneous population or colony of cells that the majority of cells might be negative [44–50]. The normality and positivity of hESC open epigenome differentiate pluripotent hESCs from any other stem cells, such as pluripotent iPS cells reprogrammed from adult cells, ESC derived from cloned embryos, and tissue-resident stem cells [14, 18, 20, 21]. Although pluripotent, the iPS cells are made from adult cells, therefore, iPS cells carry many negative repressive chromatin remodeling factors and unerasable genetic imprints of adult cells that pluripotent hESCs do not have [21,51,52]. Somatic cell nuclear transfer and factor- or small-molecule-based reprogramming are incapable of restoring a correct epigenetic pattern of pluripotent hESCs [21,51–54]. As an alternative approach to iPS cells, known neural-fate determining genes or chemicals were recently used to transdifferentiate or reprogram fibroblasts or tissues into induced adult neural cells by genetic engineering or induction with extremely low efficiencies [55–59]. Similarly, known cardiac-fate determining genes or chemicals were recently used to transdifferentiate or reprogram fibroblasts or tissues into induced adult cardiac progenitors and cardiomyocytes by genetic engineering or induction with extremely low efficiencies [60–63]. Reprogrammed somatic cells have historically been associated with abnormal gene expression, accelerated senescence, and immune-rejection following transplantation [21,51–54]. These major drawbacks have severely impaired the utility of reprogrammed or deprogrammed or direct/trans-differentiated somatic cells as viable therapeutic approaches. Although small molecules used to induce hESC lineage-specific therapy derivatives are usually safe developmental signal molecules and morphogens, it should be cautious of the small molecules used in the reverse process to generate iPS cells or trans-differentiation, which are known toxic cancerogenic chemicals with too dangerous or even lethal side effects to be used for patients [3,21,48,49,63].

## 3. TRANSFORM PLURIPOTENT HUMAN EMBRYONIC STEM CELLS INTO NEURONAL FATE-RESTRICTED THERAPY DERIVATIVES FOR CNS REGENERATION

### 3.1 Direct Induction of a Cascade of Uniform Neuronal Lineage-Specific Progression from the Pluripotent State of hESCs Using Small Molecules

The development of better differentiation strategies that permit to channel the wide differentiation potential of pluripotent hESCs efficiently and predictably to desired phenotypes is vital for realizing the therapeutic potential of hESCs. Conventional hESC differentiation procedures largely rely on the formation of multi-lineage aggregates that contain cells from all three embryonic germ layers, in part because it has been assumed that tissue and organ systems arise from the endo-, meso-, and ecto-derms. However, the nervous system and the heart are among the first tissue and organ systems formed from the cells of the ICM in embryogenesis. In fact, substantial neural and cardiac differentiation appears to occur at relatively early stages in embryonic stem cells cultivation under conditions that induce differentiation [22–26]. It is deducible that the specification of early embryonic neural and cardiac lineages may occur directly from the pluripotent hESCs, precede the germ layer formation, and subsequently influence lineage determination at later stages of the developmental continuum. Direct induction of pluripotent hESCs exclusively into a rich collection of neural- or cardiac-restricted progenies will open the door for investigating the molecular and cellular cues in directing hESC differentiation programs using effective *in vitro* model systems. These studies will permit control conditions to derive not only mature functional lineages but intermediate stem/progenitor/precursor cells for cell-based therapies.

To achieve uniformly conversion of pluripotent hESCs to a lineage-specific fate, we have employed the defined culture system capable of insuring hESC proliferation to screen a variety of small molecules and growth factors on the pluripotent state of hESCs in our recent reports. We found that pluripotent hESCs maintained under the defined culture conditions can be uniformly converted into a specific neural or cardiac lineage by small molecule induction [3,12–21]. RA was identified as sufficient to induce the specification of neuroectoderm direct from the pluripotent state of hESCs and trigger a cascade of neuronal lineage-specific progression to human neuronal progenitors (hESC-I hNuP) and neurons (hESC-I hNu) of the developing CNS in high efficiency, purity, and neuronal lineage specificity by promoting nuclear translocation of the neuronal specific transcription factor Nurr-1 [3,14,16–21] (Figs. 2, 3). Upon exposure of undifferentiated hESCs maintained in the defined culture to RA, all the cells within the colony underwent morphology changes to large differentiated cells that ceased expressing pluripotence-associated markers (e.g., Oct-4) and began expressing neuroectoderm-associated markers, but not markers associated with other lineages (Stage 1 – Human Neuroectodermal Cells) (Figs. 2,3) (Table 1). These differentiating hESCs then formed neuroblasts that were uniformly positive for β-III -tubulin in suspension (Stage 2 – Human Neuronal Progenitor Cells [hESC-I hNuP]) (Figs. 2,3). After permitting the neuroblasts to attach, β-III-tubulin- and Map-2-expressing, exuberantly neurite-bearing cells and pigmented cells began to appear with a drastic increase in efficiency (> 90%) (Stage 3 – Human Neuronal Cells in the developing CNS [hESC-I hNu]) when compared to similarly cultured cells derived from untreated EBs (<5%) (Figs. 2,3). This technology breakthrough enables neuronal lineage-specific differentiation direct from the pluripotent state of hESCs with small molecule induction, providing a much-needed *in vitro* model system for investigating molecular controls in human CNS development in embryogenesis as well as a large supply of clinical-grade human neuronal cells across the spectrum of developmental stages for tissue engineering and cell therapy against CNS disorders.

The traditional sources of engraftable human stem cells with neural potential for transplantation therapies have been multipotent human neural stem cells (hNSCs) isolated directly from the human fetal neuroectoderm or CNS [64–68]. These CNS-derived primary hNSCs are neuroepithelial-like cells that are nestin-positive and can spontaneously differentiate into a mixed population of cells containing undifferentiated hNSCs, neurons, astrocytes, and oligodendrocytes *in vitro* and *in vivo* [69–72]. These primary hNSCs can be maintained as stable cell lines that express neural stem cell markers (e.g., nestin, Sox-2, musashi) in serum-free, mitogen-supplemented media [2,67]. Upon removal of mitogens, 10–30% of these hNSCs spontaneously differentiate into cells that express the neuronal markers (e.g., β-III-tubulin, Map-2) [73,74]. The capacity of these primary hNSCs to respond to cues that might direct them towards a particular CNS neuronal subtype *in vitro* was best established in dopaminergic (DA) differentiation, producing as high as 5–10% tyrosine hydroxylase (TH)-positive ventral mesencephelon (VM) neuronal cells that expressed midbrain DA neuron markers (e.g., Nurr1, Ptx3) [75,76]. In animal models of DA dysfunction, as high as 5% of neuronal cells associated with a DA phenotype were observed [67,70,75,76]. However, cell therapy based on CNS tissue-derived hNSCs has encountered supply restriction and difficulty to use in the clinical setting due to their declining plasticity with aging and limited expansion ability, making it difficult to maintain a large scale culture and potentially restricting the tissue-derived hNSC as an adequate source for graft material [2,3]. Despite some beneficial outcomes, CNS-derived hNSCs appeared to exert their therapeutic effect primarily by their non-neuronal progenies through producing trophic and/or neuro-protective molecules to rescue endogenous host neurons, but not related to regeneration from the graft or host remyelination [2,66,67]. The small numbers of neuronal progenies generated from those engrafted hNSCs often fail to achieve the anticipated mechanism of direct reconstruction of the damaged CNS structure and circuitry [2,66,67]. So far, due to these major limitations, cell therapies based on CNS-derived hNSCs have not yielded the satisfactory results expected for clinical trials to move forward [77].

### 3.2 Neuroectoderm Specification Transforms Pluripotent hESCs into a More Neuronal Lineage-Specific Embryonic Neuronal Progenitor than the Prototypical Neuroepithelial-Like hNSCs

Alternatively, the genetically stable pluripotent hESCs proffer cures for a wide range of neurological disorders by supplying the diversity of human neuronal cell types in the developing CNS for regeneration and repair [2,3]. Therefore, pluripotent hESCs have been regarded as an ideal source to provide an unlimited supply of human neuronal cell types and subtypes for regenerating the damaged or lost nerve tissues in CNS disorders. Although neural lineages appear at a relatively early stage in differentiation, < 5% hESCs undergo spontaneous differentiation into neurons [2,3]. RA does not induce neuronal differentiation of undifferentiated hESCs maintained on feeders [2,3]. And unlike mouse ESCs, treating hESC-differentiating multi-lineage aggregates (EBs) only slightly increases the low yield of neurons [23,24]. Under conventional protocols presently employed in the field, these neural grafts derived from pluripotent cells through multi-lineage differentiation yielded neurons at a low prevalence following engraftment, which were not only insufficient for regeneration or reconstruction of the damaged CNS, but also accompanied by unacceptably high incidents of teratoma and/or neoplasm formation [2,3,5–9]. Similar to CNS-derived hNSCs, these hESC-derived secondary hNSCs are neuroepithelial-like cells that are nestin-positive and can spontaneously differentiate into a mixed population of cells containing undifferentiated hNSCs, neurons (<10%), astrocytes, and oligodendrocytes *in vitro* and *in* vivo [25,79–82]. Before further differentiation, those secondary hNSCs were tediously mechanically isolated or enriched from hESC-differentiating multi-lineage aggregates [2]. Previously, co-culturing with stromal cells or telomerase-immortalized midbrain astrocytes as well as exposing to FGF and sonic hedgehog (SHH) signaling have been used to improve the yield of β-III-tubulin- and TH-positive cells from hESC-derived hNSCs [83–85]. The signaling factors that operate along the rostrocaudal and dorsoventral axes of the neural tube to specify motor neuron fate *in vivo* have been used to direct hESCs differentiate into an early motor neuron phenotype through germ-layer induction *in vitro*, but with low efficiencies [86, 87]. Early study of these uncommitted hESC-derived hNSCs showed that the grafted cells not only yielded a small number of DA neurons (~ 0.2%) *in vivo* following transplantation, but could not acquire a DA phenotype in the lesioned brain [88]. Transplanting DA neurons pre-differentiated from these hESC-derived hNSCs *ex vivo* did not increase the yield of DA neurons in the lesioned brain [89,90]. Although a small number of motor neurons were observed following transplantation of the ESC-derived grafts into adult paralyzed rats, there was little evidence of improved behavior [86,87,91]. Similar to their CNS counterpart, the therapeutic effect of these hESC-derived hNSCs was mediated by neuroprotective or trophic mechanism to rescue dying host neurons, but not related to regeneration from the graft or host remyelination [2,3]. Growing evidences indicate that these secondary hNSCs derived from hESCs *via* conventional multi-lineage differentiation *in vitro* appear to have increased risk of tumorigenicity but not improved neurogenic potential compared to primary hNSCs isolated from the CNS tissue *in vivo*, remaining insufficient for CNS regeneration. A recent report showed that further directed differentiation of those hESC-derived hNSCs into floor-plate precursors of the developing midbrain appeared to increase the efficiency of DA neuron engraftment in Parkinson’s disease models, further suggesting that the poor *in vivo* performance of those nestin-positive neuroepithelial-like hNSCs derived from hESCs *in vitro* was due to incomplete neuronal lineage specification [3,10]. Development of a well-controlled strategy for efficiently committing hESCs into a more specific neuronal lineage in high purity and large quantity is vital to harnessing the therapeutic potential of pluripotent hESCs for CNS repair.

Unlike the two prototypical hNSCs, hESC-I hNuPs, which have acquired a neuroectodermal identity through RA induction of pluripotent hESCs *in vitro*, did not express the canonical hNSC markers [e.g., nestin], but assumed uniformly strong expression and nuclear localization of the neuronal specific transcriptional factor Nurr-1 [3,17,18]. Under neuronal differentiation conditions, hESC-I hNuPs yielded exclusively neurons that expressed neuronal markers with a drastic increase in efficiency (~ 95%) when compared to the yields of β-III-tubulin-positive neurons differentiated under similar conditions from hESC-derived hNSCs (~ 6%) or CNS-derived hNSCs (~ 13%) [3,17–19]. These *in vitro* neuroectoderm-derived hESC-I hNuPs yielded neurons efficiently and exclusively, as they did not differentiate into other neural cell types such as glial cells, suggesting that they are a novel more neuronal lineage-specific embryonic neuronal progenitor than the prototypical neuroepithelial-like hNSCs. MiRNAs act as the governors of gene expression networks, thereby modify complex cellular phenotypes in development or disorders [16,19]. MiRNAs play a key role in regulation of ESC identity and cell lineage in mouse and human ESCs [16,19]. MiRNA expression profiling using microarrays is a powerful high-throughput tool capable of monitoring the regulatory networks of the entire genome and identifying functional elements in hESC development [16,19]. Genome-scale profiling of microRNA (miRNA) differential expression showed that the expression of pluripotence-associated hsa-miR-302 family was silenced and the expression of Hox miRNA hsa-miR-10 family that regulates gene expression predominantly in neuroectoderm was induced to high levels in those hESC-derived neuronal progenitors [16,19]. Following transplantation, they engrafted widely and yielded well-dispersed and well-integrated human neurons at a high prevalence within neurogenic regions of the brain [18,19]. These studies suggest that these hESC neuronal derivatives have acquired a neuronal lineage-specific identity by silencing pluripotence-associated miRNAs and inducing the expression of miRNAs linked to regulating human CNS development to high levels, therefore, highly neurogenic *in vitro* and *in vivo* [3,16–21]. Therefore, neuroectoderm specification of pluripotent hESCs produces an engraftable human embryonic neuronal progenitor in high purity and large supply with adequate neurogenic potential for scale-up CNS regeneration [3,16–21] (Fig. 2). Under protocols presently employed in the field, hESC-derived cellular products consist of a heterogeneous population of mixed cell types, including fully differentiated cells, high levels of various degrees of partially differentiated or uncommitted cells, and low levels of undifferentiated hESCs, posing a constant safety concern when administered to humans [2,3]. Novel lineage-specific differentiation approach by small molecule induction of pluripotent hESCs not only provides a model system for investigating human embryogenesis, but also dramatically increases the clinical efficacy of graft-dependent repair and safety of hESC-derived cellular products. Thus, it offers an adequate human neurogenic cell source in high purity and large quantity for CNS tissue engineering and developing safe and effective stem cell therapy to restore the normal nerve tissue and function in a wide range of neurological disorders.

### 3.3 Turning Pluriptoent hESCs into a Large Supply of Plastic CNS Derivatives for Modeling Human CNS Development, Tissue Engineering, and Cell Therapy

Understanding the much more complex human embryonic development has been hindered by the restriction on human embryonic and fetal materials as well as the limited availability of human cell types and tissues for study. In particular, there is a fundamental gap in our knowledge regarding the molecular networks and pathways underlying the CNS and heart formation in human embryonic development. The enormous diversity of human somatic cell types and the highest order of complexity of human genomes, cells, tissues, and organs among all the eukaryotes pose a big challenge for characterizing, identifying, and validating functional elements in human embryonic development in a comprehensive manner. Derivation of hESCs provides a powerful *in vitro* model system to investigate the molecular controls in human embryonic development as well as an unlimited source to generate the diversity of human cell types and subtypes across the spectrum of development stages for repair. Development and utilization of hESC models of human embryonic development will facilitate rapid progress in identification of molecular and genetic therapeutic targets for the prevention and treatment of human diseases.

After neuronal induction during early mammalian embryogenesis, neuroectodermal cells form the neural plate that develops into the neural tube. Subsequent development, together with vesiculation within the tube, gives rise to the brain and spinal cord of the CNS. The complexity in generating the enormous diversity of neuronal cell types is best illustrated in the development of mammalian telencephalon [92,93]. The most rostral region of the neural tube, the prosencephalon, divides into the telencephalon and diencephalon. The dorsal region of the telencephalon gives rise to the cerebral cortex, which comprises the neucortex, piriform cortex and hippocampus, while the ventral telencephalon differentiates into the basal ganglia. The dorsoventral and rostrocaudal identities and subsequent specification of progenitors are established by diffusible morphogens, including Fgf, Shh, Bmp, Wnt, Nodal, and Notch proteins, through regional patterning and activation of transcription factors [94]. The key steps of neurogenesis include neuronal commitment of NSCs, the subtype specification of intermediate neuronal progenitors, postmitotic precursors, and mature neurons [92–94] (Table 1). Developing multi-cellular models of human CNS development using hESCs will contribute tremendously to our knowledge regarding molecular neurogenesis in human embryonic development, and thereby, aid the formulation of more optimal cell-based therapeutic strategies for CNS repair.

Recent development in innovative small molecule direct induction approach renders a cascade of neuronal lineage-specific progression directly from the pluripotent state of hESCs, providing much-needed *in vitro* model systems for investigating the human CNS development in embryogenesis [3,16–21] (Fig. 2, Fig. 3). This technology breakthrough not only opens the door for further identification of the developmental networks in human embryonic neurogenesis in a comprehensive manner, but also offers means for small-molecule-mediated direct control and modulation of the pluripotent fate of hESCs when deriving an unlimited supply of neuronal cell types and subtypes for regenerative medicine [3,16–21]. Advances in large-scale profiling of developmental regulators in high-resolution have provided powerful genome-wide high-throughput approaches that lead to great advances in our understanding of the global phenomena of human embryogenesis. Studies to profile novel hESC models of human embryonic neurogenesis using genome-wide approaches, including employing chromatin/nucleosome-immunoprecipitation-coupled DNA microarray analysis (ChIP/NuIP-chip) and miRNA mapping, have revealed molecular controls and the underlying mechanisms in hESC neuronal lineage specification [16,19–21]. Such genome-wide high-resolution maps will generate comprehensive knowledge of developmental regulators and networks underlying hESC neuronal cell type and subtype specification for systems biology approaches and network models of human embryogenesis. Unveiling developmental networks during human embryonic neurogenesis using novel hESC models will contribute tremendously to our knowledge regarding molecular embryogenesis in human development, thereby, reveal potential therapeutic targets and aid the development of more optimal stem-cell-mediated therapeutic strategies for the prevention and treatment of CNS disorders. The outcome of such research programs will potentially shift current research to create new scientific paradigms for developmental biology and stem cell research.

Standard stem cell differentiation protocols involve cultivation in 2-dimensional (2D) settings, whereas *in vivo* organogenesis requires a 3-dimensional (3D) setting to provide the spatial and temporal controls of cell differentiation necessary for the formation of functional tissues [95–97]. The traditional methods of 2D culture often result in unpredictable stem cell function and behavior *in vivo* following transplantation [95–97]. Because of interspecies differences, conventional studies using animal models are often poor predictors of human efficacy and safety. Animal models are xeno-hosts for transplantation of human cells, not ideal for testing the safety and efficacy of therapeutic outcomes of human stem cells. Large primate models are very costly and often taken years to obtain results. In addition, the results of animal studies can be highly variable and difficult to reproduce, making them unreliable as benchmarks for decisions on human clinical trials. Developing strategies for complex 3D multi-cellular models of human embryogenesis and organogenesis will provide a powerful tool that enables analysis under conditions that are tightly regulated and authentically representing the *in vivo* spatial and temporal patterns. It will go beyond flat biology to increase the biological complexity of human-based *in vitro* models and assays to mimic the *in vivo* human organ systems and functions, which are controllable, reproducible, and scalable, and can be monitored and validated against responses on multiple hierarchical levels.

Advancements in micro- and nano-fabrication techniques offer the possibility for highly reproducible mass-fabrication of systems with complex geometries and functionalities [96, 98]. Tissue specific extracellular matrix (ECM) gels can now create structures and surfaces with defined shapes that can be used to position cells and tissues, control cell shape and function, and create highly structured 3D culture microenvironments [96–98]. Hydrogels are excellent scaffolding materials for repairing and regenerating a variety of tissues because they can provide a highly swollen 3D environment similar to soft tissues [99,100]. Bio-mimetic modification of hydrogels as tissue engineering scaffolds has emerged as an important strategy to modulate specific cellular responses for the incorporation of key biofunctions of natural ECMs to provide valuable insight into the regulation of cell function and developmental processes in tissue- and organ-specific differentiation and morphogenesis [101,102]. In addition, the recently developed NanoCulture Plate has a precisely engineered pattern (microsquare or microhoneycomb) that promotes cells to form uniform spheroids that are highly reproducible.

Realizing the developmental and therapeutic potential of hESCs has been hindered by conventional approaches for generating functional cells from pluripotent cells through multi-lineage differentiation in 2D culture, which is uncontrollable, inefficient, instable, highly variable, difficult to reproduce and scale-up [2,3]. Development and utilization of multi-cellular 3D human embryonic models using hESCs will provide an authentic and reliable *in vitro* tool targeted for rapid and high fidelity safety and efficacy evaluation of human therapeutic candidates and products, and thus reduce the reliance on animal models to test potential therapeutic strategies and lead to advances in technologies used in the regulatory review. It will dramatically increase the overall turnover of investments in biomedical sciences and facilitate rapid progress in identification of therapeutic targets and approaches for the prevention and treatment of human diseases. Combining innovations in establishing highly efficient hESC neuronal lineage-specific differentiation protocol with the advancements in 3D culture microenvironments to develop the multi-cellular 3D models of the human CNS will provide much-needed *in vitro* tools for biomedical research. Under 3D neuronal subtype specification conditions, these hESC-derived neuronal cells by small molecule induction further proceeded to express subtype neuronal markers associated with ventrally-located neuronal populations, such as DA neurons and motor neurons, demonstrating their potential for neuron regeneration *in vivo* as stem cell therapy to be translated to patients in clinical trials [19].

Recent studies found that pluripotent hESCs maintained under the defined culture conditions can be uniformly converted into a specific neural or cardiac lineage by small molecule induction [3,12–21] (Fig. 2). This technology breakthrough enables well-controlled generation of a large supply of neuronal lineage-specific progenies across the spectrum of developmental stages direct from the pluripotent state of hESCs with signal molecules, providing unlimited source of engraftable hESC neuronal therapy derivatives in high purity, large scale, and neuronal-lineage specificity with adequate neurogenic capacity for regenerating the damaged or lost CNS structure and circuitry in a wide range of neurological disorders. Further assessment of their potential in disease models and 3D CNS models will offer critical insights into therapeutic strategies against CNS disorders as well as provide preclinical evidences of safety and efficacy for translating to patients in clinical trials. The availability of human neuronal progenitors and neuronal cells in high purity and large quantity with adequate neurogenic potential will facilitate CNS tissue-engineering and accelerate the development of safe and effective cell-based therapy against a wide range of neurological disorders that so far remain incurable, including Parkinson’s disease, Alzheimer disease, ALS, spinal muscular atrophy, stroke, brain and spinal cord injuries.

## 4. TRANSFORM PLURIPOTENT HUMAN EMBRYONIC STEM CELLS INTO CARDIAC FATE-RESTRICTED THERAPY DERIVATIVES FOR MYOCARDIUM REGENERATION

### 4.1 A Well-Controlled Efficient Approach for hESC Cardiac Lineage-Specific Progression Direct from the Pluripotent Stage towards Beating Cardiomyocytes by Small-Molecule Induction

Cardiovascular disease (CVD) is a major health problem and the leading cause of death in the Western World. So far, the lack of a clinically-suitable human cardiomyocyte source with adequate myocardium regenerative potential has been the major setback in regenerating the damaged human heart, either by endogenous cells or by cell-based transplantation or cardiac tissue engineering [3,12,13,78,103,104]. In the adult heart, the mature contracting cardiac muscle cells, known as cardiomyocytes, are terminally differentiated and unable to regenerate. Damaged or diseased cardiomyocytes are removed largely by macrophages and replaced by scar tissue. Although cell populations expressing stem/progenitor cell markers have been identified in postnatal hearts, the minuscule quantities and growing evidences indicating that they are not genuine heart cells have caused skepticism if they can potentially be harnessed for cardiac repair [78,105–110]. There is no evidence that stem/precursor/progenitor cells derived from other sources, such as bone marrow, cord blood, umbilical cord, fat tissue, or placenta, are able to give rise to the contractile heart muscle cells following transplantation into the heart [3,78,104].

The heart is the first organ formed from the cells of the ICM or epiblast of the blastocyst in early embryogenesis. During vertebrate embryogenesis, the major morphogenetic and regulative events that control myocardial progenitor cell differentiation into cardiomyocytes include four sequential but overlapping processes: specification of the cardiogenic mesoderm, determination of the bilaterally symmetric heart fields, patterning of the heart field, and finally cardiomyocyte differentiation and formation of the heart tube [111,112] (Table 2). In addition, endocardial and extracardiac cell populations, including smooth muscle cells, endothelial cells, and connective tissue elements, contribute to the fully functional mature heart. The development of the heart appears to be regulated by complex positive and negative signaling networks involving members of the Bmp, Shh, Fgf, Wnt, Nodal, and Notch proteins [108,113]. The heart forms from two distinct progenitor cell populations or heart fields that segregate from a common progenitor at gastrulation [113,114]. The primary and second heart fields can be distinguished by the expression of specific transcription factors and signaling molecules. For example, Tbx5 and Hand1 mark the primary heart field, whereas Hand2, Isl1, Tbx1, Foxh1, Mef2c, and Fgf8/10 mark the secondary heart field, although some cardiac regulatory genes, such as Nkx2.5, are expressed in both heart fields [108,113,114]. Developing cellular models of human embryonic heart formation will reveal the biological pathways and molecular targets that control cardiogenesis in human embryonic development, thereby, aids identification of molecular and genetic therapeutic targets for the prevention and treatment of heart disease and failure [3,115].

Due to the prevalence of CVD worldwide and acute shortage of donor organs or adequate human myocardial grafts, there is intense interest in developing hESC-based therapy for heart disease and failure. In hESC-differentiating multi-lineage aggregates (EBs), only a very small fraction of cells (< 4 %) spontaneously differentiate into cardiomyocytes [3,26]. Immune-selection, co-culturing, and morphogens have been used to isolate and enrich populations of immature cardiomyocytes from hESC-differentiating EBs [26,116–119]. Enriched hESC-derived cardiomyocytes could generate small grafts and function as the biological pacemaker in animal infarcted models [27]. Although such hESC-derived cardiomyocytes can partially remuscularize the injured heart and attenuate the progression of heart failure in animal models of acute myocardial infarction up to 12 weeks, equivalent to perhaps a few months in humans, the grafts generated by cell transplantation have been small and insufficient to restore heart function or to alter adverse remodeling of chronic infarcted models following transplantation [120–123]. Thus, developing novel strategies to channel the wide differentiation potential of pluripotent hESCs exclusively and predictably to a cardiac phenotype is vital to harnessing the power of hESC biology for cardiac repair.

Recent studies found that formulation of minimal essential defined conditions for hESCs rendered small molecule NAM sufficient to induce the specification of cardiomesoderm direct from the pluripotent state of hESCs by promoting the expression of the earliest cardiac-specific transcription factor Csx/Nkx2.5 and triggering progression to cardiac precursors and beating cardiomyocytes with high efficiency [3,12–16,21] (Figs. 2,4). Upon exposure of undifferentiated hESCs maintained in the defined culture to NAM, all the cells within the colony underwent morphology changes to large differentiated cells that down-regulated the expression of pluripotence-associated markers (e.g., Oct-4, Sox-2) and began expressing the earliest marker for heart precursor (e.g., Nkx2.5, alpha-actinin), but not markers associated with other lineages (Stage 1 --- Human Cardiomesodermal Cells) (Figs. 2,4) (Table 2).

These differentiating hESCs then formed cardioblasts that uniformly expressed Nkx2.5 in suspension (Stage 2 --- Human Cardiac Precursor Cells) (Figs. 2,4). After permitting the cardioblasts to attach and further treating them with NAM, beating cardiomyocytes began to appear after withdrawal of NAM with a drastic increase in efficiency when compared to similarly cultured cells derived from untreated EBs (Stage 3 --- Human Cardiomyocytes) (Figs. 2, 4). Cells within the beating cardiospheres expressed markers characteristic of cardiomyocytes [3,12,15] (Figs. 2,4). Electrical profiles of the cardiomyocytes confirmed their contractions to be strong rhythmic impulses reminiscent of the p-QRS-T-complexes seen from body surface electrodes in clinical electrocardiograms [15]. This technology breakthrough enables cardiac lineage-specific differentiation direct from the pluripotent state of hESCs with small molecule induction, providing a much-needed *in vitro* hESC model system for investigating molecular controls in human embryonic heart formation as well as a large supply of clinical-grade human cardiomyocyte precursors and cardiomyocytes for myocardial tissue engineering and cell therapy against heart disease and failure. NAM appeared to induce global histone deacetylation, significant down-regulation of the expression of active chromatin remodeling factors associated with a pluripotent state, and nuclear translocation of the class III NAD-dependent histone deacetylase SIRT1 [16,21]. This observation suggests that NAM triggers the activation of SIRT1 and NAD-dependent histone deacetylation that lead to global chromatin silencing yet selective activation of a subset of cardiac-specific genes, and subsequently cardiac fate determination of pluripotent hESCs [16,21]. Further unveiling the neucleoprotein complex regulation in hESC cardiac lineage specific progression towards cardiomyocytes mediated by SIRT1 will provide critical understanding to the molecular mechanism underlying human embryonic cardiogenesis, thereby aid the development of more effective and safe stem cell-based therapeutic approaches in the heart field.

### 4.2 Cardiac Lineage-Specific Differentiation of Pluripotent hESCs by Small Molecule Induction Opens the Door to Model the Human Heart Formation

Advances in human miRNA expression microarrays, ChIP-chip, and chromatin-immunoprecipitation-combined second-generation high-throughput sequencing (ChIP-seq) have provided powerful genome-wide, high-throughput, and high resolution techniques that lead to great advances in our understanding of the global phenomena of human embryonic developmental processes using hESCs [16,19–21,124–126]. ChIP-seq is a most recently developed technique for genome-wide profiling of DNA-binding proteins, histone or nucleosome modifications using next-generation deep DNA sequencing technology [21,125, 126]. ChIP-seq offers higher resolution, less noise and greater coverage than its array-based predecessor ChIP-chip, and has become an indispensable tool for studying gene regulation and epigenetic mechanisms in development [21,125,126]. However, without a practical strategy to convert pluripotent cells direct into a specific lineage, previous studies are limited to profiling of hESCs differentiating multi-lineage aggregates, such as EB that contain mixed cell types of endoderm, mesoderm, and ectoderm cells or a heterogeneous population of EB-derived cardiac or cardiovascular cells that contain mixed cell types of cardiomyocytes, smooth muscle cells, and endothelial cells [21,124,125]. Those previous reports have not achieved to utilize high-throughput approaches to profile one particular cell type differentiated from hESCs, such as neurons or cardiomyocytes [21,124,125]. Their findings have been limited to a small group of genes that have been identified previously in non-human systems, and thus, have not uncovered any new regulatory pathways unique to humans [21,124,125]. Due to the difficulty of conventional multi-lineage differentiation approaches in obtaining the large number of purified cells typically required for ChIP-seq experiments, studies to reveal the mechanism in hESC differentiation remain lacking [21], though genome-wide mapping of histone modifications and chromatin-associated proteins have already begun to reveal the mechanisms in mouse ESC differentiation [127].

Recent development in innovative small molecule direct induction approach renders a cascade of neuronal or cardiac lineage-specific progression directly from the pluripotent state of hESCs, providing much-needed *in vitro* model systems for investigating the human CNS development and heart formation in embryogenesis [3,12–21] (Figs. 2–4). Such *in vitro* hESC model systems enable direct generation of large numbers of high purity hESC neuronal or cardiomyocyte derivatives required for ChIP-seq analysis to reveal the mechanisms responsible for regulating the patterns of gene expression in hESC neuronal or cardiomyocyte specification [21]. This technology breakthrough not only opens the door for further identification of the developmental networks in human embryonic cardiogenesis in a comprehensive manner, but also offers means for small-molecule-mediated direct control and modulation of the pluripotent fate of hESCs when deriving an unlimited supply of human cardiac cells and cardiomyocytes for regenerative medicine. Profiling novel hESC models of human embryonic cardiogenesis using genome-wide approaches has begun to reveal molecular controls and the underlying mechanisms in hESC cardiac specification [16]. Such genome-wide high-resolution maps will generate comprehensive knowledge of developmental regulators and networks underlying hESC cardiomyocyte specification for systems biology approaches. Unveiling developmental networks during human embryonic heart formation using novel hESC models will contribute tremendously to our knowledge regarding molecular cardiogenesis in human embryonic development, thereby, reveal potential therapeutic targets and aid the development of more optimal stem-cell-mediated therapeutic strategies for the prevention and treatment of heart diseases. The outcome of such research program will potentially shift current research to create new scientific paradigms for developmental biology and stem cell research.

Development and utilization of complex 3D multi-cellular hESC models of human embryonic heart formation will provide a powerful tool that enables analysis under conditions that are tightly regulated and authentically representing the *in vivo* spatial and temporal patterns of the heart, and thus reduce the reliance on animal models to test potential therapeutic strategies against CVD. Recent technology breakthrough enables well-controlled efficient generation of a large supply of cardiac lineage-specific progenies across the spectrum of developmental stages direct from the pluripotent state of hESCs for innovations to develop the multi-cellular 3D human embryonic model that can replicate the aspects of the human heart [3,12–16,21]. Heart formation requires cardiac cellular constituents and cardiovascular architectural support. The hESC-derived cardiac elements resemble the cardiac cells in human embryogenesis; therefore, they have the intrinsic potential to form human contractile heart muscle as well as the cardiovascular structure with 3D geometry and vasculature of the heart. The hESC-derived multi-cellular heart models will be able to represent cellular, functional and structural characteristics of the human heart to increase the biological complexity of human-based *in vitro* models and assays to mimic the *in vivo* structure, behavior, and function of the human organs. Such studies will provide a powerful tool targeted for rapid and high fidelity safety and efficacy evaluation of human therapeutic candidates and human cell therapy products against heart diseases, and thus lead to advances in technologies used in the regulatory review in the heart field.

### 4.3. Human Embryonic Stem Cell Cardiomyocyte Derivatives for Heart Regeneration — the Vital Source for Myocardial Tissue Engineering and Myocardium Repair

To date, the lack of a suitable human cardiomyocyte source with adequate myocardium regenerative potential has been the major setback for myocardial tissue engineering as well as for developing safe and effective cardiac cell therapy. Novel approach using small molecule direct induction of pluripotent cells into cardiac precursors and cardiomyocytes offers the benefits in efficiency, stability, safety, and scale-up production over existing conventional approaches [3,12–16] (Figs. 2,4). Such technology breakthroughs in hESC research enable *de novo* derivation of clinically-suitable stable hESC lines from human blastocysts and direct conversion of such pluripotent hESCs into a large supply of clinical-grade functional human cardiomyocyte precursors and cardiomyocytes to be translated to patients for mending the damaged heart [3,12,13]. The availability of human cardiomyocyte derivatives in high purity and large quantity with adequate potential for myocardium regeneration will facilitate myocardial tissue-engineering and accelerate the development of safe and effective cell-based therapy for heart disease and failure that affect millions of survivors and so far have no cure [12,13]. It makes heart disease and failure possible to be the first major health problem to be resolved by clinical translation of the advances of hESC research [12,13].

Current cell delivery methods to the damaged heart, by injection of cells either directly into the infarcted region or via the coronary circulation, are inefficient [78,103,104]. In addition, arrhythmogenesis is a potential risk in cell-based cardiac repair [78,103,104]. So far, the need to regenerate or repair the damaged heart muscle (myocardium) has not been met by adult stem cell therapy, either endogenous or via cell delivery [78,103,104]. Heart transplantation with the donor organ has been the only definitive treatment for end-stage heart failure. For millions living with the damaged heart, there is no alternative definitive treatment available at present time. And there is an acute shortage of donor organs for patients who need the heart transplantation. For end-stage heart failure, stem/progenitor-cell-mediated cellular regenerative approach cannot be used as an alternative approach to heart transplantation. Those shortcomings provoke developing the technology of using hESC-derived cardiac elements to reconstitute the human hearts as the replacement organ in order to provide alternative treatment options to donor-based heart transplantation. In case of successful heart transplantation from suitable donor organ, it requires life-long immune suppression that is often associated with serious side effects. The hESCs and their derivatives are considerably less immunogenic than adult tissues [103,104]. It is also possible to bank large numbers of human leukocyte antigen isotyped hESC lines so as to improve the likelihood of a close match [103,104]. Therefore, making the heart from hESC-derived cardiac elements will not only provide a 3D human heart model that authentically represents the *in vivo* organ system and function for understanding human embryonic heart formation, but also will have tremendous potential to translate to clinical studies for organ-replacement therapy to meet some of the critical medical challenges resulted from shortage of donor organs and immune rejection in heart transplantation [12,13].

Cardiomyocytes contribute to most of the structural volume of the heart. The relative simplicity in development and maturation of the embryonic heart makes it also possible to be the first organ to be reconstituted from hESCs, the *in vitro* representation of the ICM/epiblast. Establishing a controllable differentiation route to efficiently generate a large supply of human cardiac elements from hESCs will make it become feasible to reconstruct the human beating heart in 3D with appropriate cellular constituents and cardiovascular architecture [3,12,13,15]. The hESC-derived cardiac elements *in vitro* may resemble the cardiac cells in human embryogenesis *in vivo*; therefore, they have the potential to form a perfect match to the human beating heart [12,13]. Using hESC cardiac derivatives to reconstitute the 3D human heart that reflects the biological complexity and microenvironment niche of the *in vivo* human heart and function will facilitate rapid progress in the identification of molecular and genetic therapeutic targets for prevention and treatment of CVD. Such studies will lead to reconstituting fully competent human hearts in 3D from hESC cardiac derivatives to meet the medical need of replacement organs for end-stage heart disease and failure, a major leap in regenerative medicine. It will provide groundbreaking technology platform for tissue and organ reconstitution from hESC-derived somatic elements, innovating in regenerative medicine that will have a tremendous impact on biomedical sciences and the healthcare industry.

## 5. FUTURE PROSPECTIVES

Human stem cell therapy derivatives are extremely attractive for therapeutic development because they have direct pharmacologic utility in clinical applications, unlike any cells originated from animals and other lower organisms that are only useful as research materials. The human stem cell is emerging as a new type of pharmacologic agent of cellular entity in cell-based regenerative medicine, because human stem cell therapy derivatives have the potential for human tissue and function restoration that the conventional drug of molecular entity lacks. The ability of a human stem cell, by definition, to both self-renew and differentiation makes it a practically inexhaustible source of replacement cells for many devastating or fatal diseases that have been considered as incurable, such as neurodegenerative diseases and heart diseases. The pharmacologic activity of human stem cell therapy derivatives is measured by their extraordinary cellular ability to regenerate the tissue or organ that has been damaged or lost. In this regard, the pharmacologic utility of human stem cells cannot be satisfied only by their chaperone activity, if any, to produce trophic or protective molecules to rescue existing endogenous host cells that can simply be achieved by a small molecule or a drug of molecular entity. There is a large unmet healthcare need to develop hESC-based stem cell therapies to provide optimal regeneration and reconstruction treatment options to restore normal tissues and function. Clinical applications of hESC therapy derivatives provide the right alternative for many incurable diseases and major health problems that the conventional mode of drugs and treatments cannot. Recent advances and technology breakthroughs in hESC research have overcome some major obstacles in bringing hESC therapy derivatives towards clinical applications, including establishing defined culture systems for *de novo* derivation of clinically-suitable stable hESC lines from human blastocysts that have never been contaminated by animal cells and proteins, and direct conversion of such pluripotent hESCs into a large supply of clinical-grade functional human neuronal or cardiomyocyte therapy derivatives to be translated to patients for CNS or heart repair [3,12–21]. Without an understanding of the essential developmental components for sustaining hESC pluripotence and self-renewal, hESC lines are at risk for becoming unhealthy and unstable after prolonged culturing under animal feeders, feeder-conditioned media, or artificially-formulated chemically-defined conditions [3,128]. Resolving minimal essential requirements for sustaining embryonic pluripotence allows all poorly-characterized and unspecified biological additives, components, and substrates in the culture system, including those derived from animals, to be removed, substituted, or optimized with defined human alternatives for *de novo* derivation and long-term maintenance of GMP-quality xeno-free stable hESC lines and their human therapy derivatives [3,12]. Formulation of minimal essential defined conditions renders pluripotent hESCs be directly and uniformly converted into a specific neural or cardiac lineage by small signal molecule induction [3,12–21]. Such milestone advances and medical innovations in hESC research enable generation of a large supply of high purity clinical-grade hESC neuronal and heart muscle cell therapy products as powerful cellular medicines that can offer pharmacologic utility and capacity for CNS and heart regeneration that no conventional drug of molecular entity can. Currently, these hESC neuronal and cardiomyocyte therapy derivatives are the only available human cell sources with adequate capacity to regenerate neurons and contractile heart muscles, vital for CNS and heart repair in the clinical setting. The availability of human neuronal and cardiomyocyte therapy derivatives in high purity and large quantity with adequate potential for CNS and myocardium regeneration will facilitate CNS and myocardial tissue-engineering and accelerate the development of safe and effective cell-based therapy to resolve these major health problems. Further improving policy making and funding situation for hESC research would open up a new dimension of cell therapy-based future medicine to provide new medical treatments for many devastating and life-threatening diseases and injuries. Transforming pluripotent hESCs into fate-restricted therapy derivatives dramatically increases the clinical efficacy of graft-dependent repair and safety of hESC-derived cellular products, bringing cell-based regenerative medicine to a turning point.

## Figures and Tables

**Table 1:** Neural developmental stage markers

Pluripotent hESCs	Neuroectoderm cells	Neuronal precursors	Neurons	Glial cells	Non-neural cells
Oct-4 (POU5 transcription factor)	HNK-1 (human natural killer antigen-1)	Sox-2	Nurr1, β-III-tubulin (TUJ1), Map-2, NeuN (Neuron nuclear antigen), 70 KDa NF, 160 KDa NF (Neurofilament)	GFAP (astrocyte)	Nkx2.5/Csx (Cardiac-specific homeobox), GATA-4, MEF2c (myocyte enhancer factor 2c) (cardiomyocyte)
SSEA-4 (Stage-specific embryonic antigen)	AP2 (transcription factor)	Musashi (RNA-binding protein)	Lis1, DCX (doublecortin), DCLK (centrosome and microtubule associated proteins) (immature neuron)	ETT2 (astrocyte)	Hand 1, Hand 2 (heart and neural crest derivatives expressed transcript 1, 2) (cardiomyocyte)
Tra-1-60 (Tumor rejection antigen)	NCAM (polysialic acid neural cell adhesion molecule)	CD133 (prominin-1)	TUC4 (CRMP4), PSA-NCAM, Calretinin (immature neuron)	O4 (oligodendrocyte)	Tbx 1, Tbx 5, Tbx 20 (T-box 1, 5, 20), Isl1 (insulin gene enhancer protein), PITX2 (paired-like homeodomain transcription factor 2) (cardiomyocyte)
Tra-1-81 (Tumor rejection antigen)	TrkC (Tyrosine kinase C)	Ngn1,2 (Neurogenin1, 2) (proneural)	DLX1, DLX2, Mash1, Nkx2.1, Gsh2, EMX2, (Intrinsic programme)	CNPase (oligodendrocyte)	VE-cadherin (Vascular Endothelial Cell)
Alkaline Phosphatase (Tra-2-54)	Nurr1	Mash1 (mammalian achaete-scute homo-logue 1 (proneural)	E2F1 (transcription factor), Eph-ephrin signaling (EphB1-3, A4, A7), SHH (Sonic hedgehog signaling), Wnt signaling (Intrinsic programme)	MBP (oligodendrocyte)	VWF (von Willebrand factor) (blood vesicle)
Nanog (Homeobox Transcription factor)		Nurr1 (orphanNuclearHormonereceptor)	Pax6 (paired-domain 6), Tlx (orphan nuclear receptor), (dorsal-ventral patterning of the telencephalon, cortical neurons, olfactory bulb interneurons)	RIP (oligodendrocyte)	Smoothelin-A/B, SM22alpha, h1-calponin (smooth muscle)
Sox-2 (Sex determining region Y-box2)		Nestin (intermediate filament)	TH (Tyrosine hydroxylase), DAP (dopamine transporter), Nurr1, Lmx1, Msx1, Pitx3, En1/En2 (dopaminergic neuron)	GalC (oligodendrocyte)	AFP (A-fetopretein), Albumin (liver)
		Vimentin	HB9, Lim3, Islet1, Lhx3 (motor neuron)	Olig2 (oligodendrocyte transcription factor 2)	Pdx1, Insulin (beta cell)
			Glutamate, Serotonin (5-HT, 5-hydroxytryptamine), ACh (acetylcholine), NA (noradrenaline), DA (dopamine), NO (nitric oxide) (Transmitter)	Notch signaling	CCSP, Sca1 (lung)
			γ-aminobutyric Acid, GABA receptor, (GABAergic neuron) GAD (glutamate decarboxylase), Glutamate receptor, AMPAR (alpha-amino-3-hydroxy-5-methyl-4-isoxazole-propionic acid receptor), NMDAR (N-methyl-D-aspartate receptor) (Glutamatergic neuron)		Runx2, Runx3, Osterix, Sox9 (bone) Myogenin, MyoD (Skeletal Muscle)

**Table 2:** Cardiac developmental stage markers

Pluripotent hESCs	Cardiomesoderm Cells	Cardiomyocyte Precursors	Cardiomyocytes	Cardiovascular Cells	Non-Cardiac Cells
Oct-4 (POU5 transcription factor)	Nkx2.5/Csx	Nkx2.5/Csx	Nkx2.5/Csx (NK2 transcription factor related, locus 5/ Cardiac-specific homeobox)	VE-cadherin (Vascular Endothelial cell)	HNK1, AP2, NCAM, TrkC (neuroectodermal cell)
SSEA-4 (Stage-specific embryonic antigen)	GATA-4	GATA-4 (GATA binding protein 4)	GATA-4	VWF (von Willebrand factor) (blood vesicle)	Sox-2, Musashi, CD133, Nestin (neural stem cell)
Tra-1-60 (Tumor rejection antigen)	MESP1 (mesoderm posterior 1)	alpha-actinin	MEF2c (RSRF) (myocyte enhancer factor 2c, related to serum response factor)	Smoothelin-A/B (smooth muscle)	β-III-tubulin (TUJ1), Map-2, 70 KDa NF,160 KDa NF, NeuN, Pax6 (Neurons)
Tra-1-81 (Tumor rejection antigen)	MESP2 (mesoderm posterior 2)	Igfbp5 (insulin-like growth factor binding protein 5)	Hand 1, Hand 2 (heart and neural crest derivative expressed transcript 1, 2; the bHLH transcription factor)	SM22alpha, h1-calponin (smooth muscle)	TH (Tyrosine hydroxylase), DAP (dopamine transporter), Nurr1, Lmx1, Msx1, Pitx3, En1/En2 (dopaminergic neuron)
Alkaline Phosphatase (Tra-2-54)	alpha-actinin	Pdgfra (PDGF receptor α)	Tbx 1, Tbx 5, Tbx 20 (T-box 1, 5, 20), Isl1 (insulin gene enhancer protein, a LIM homeodomain transcription factor), PITX2 (paired-like homeodomain transcription factor 2),	Myocardin, SRF, SM-MHC, SM-actin (smooth muscle)	HB9, Lim3, Islet1, Lhx3 (motor neuron)
Nanog (Homeobox Transcription factor)		Odz4 (trans-membrane protein)	Foxp4, Foxh1 (fork-head box P4, H1), Cal (Csx-associated LIM pretein), Irx4 (Iroquois homeobox gene 4), HOP (homeodomain-only protein)	Isl1, Flk1, CD31 (Vascular Endothelial cell)	GFAP, ETT2 (astrocyte), CNPase, O4, MBP, RIP, GalC, Olig2 (oligodendrocyte)
Sox-2 (Sex determining region Y-box 2)		Tnc (tenascin C, matrix protein)	Fgf 8, Fgf10 (fibroblast growth factor 8, 10)		AFP (A-fetopretein), Albumin (liver)
		Pbx3 (homeodomain transcription factor)	SRF (serum response factor), ANP (atrial natriuretic peptide), CARP (cardiac ankyrin repeat protein)		Pdx1, Insulin (beta cell)
		Isl1 Fgf10	alpha-actinin, Myocardin Connexin 40, 43		CCSP, Sca1 (lung) Runx2, Runx3, Osterix, Sox9 (bone)
		Bmp2/ Smad1	MHC (Cardiac Myosin heavy Chain), cTnT (cardiac troponin T)		Myogenin, MyoD (Skeletal Muscle)

## ABBREVIATIONS

2/3D: 2/3-Dimensional
ChIP/NuIP-chip: Chromatin/Nucleosome-Immunoprecipitation Coupled DNA Microarray Analysis
ChIP-seq: Chromatin-Immunoprecipitation-Combined Second-Generation High-Throughput Sequencing
CNS: Central Nervous System
CVD: Cardiovascular Disease
DA: Dopaminergic
EB: Embryoid Body
ECM: Extracellular Matrix
EMA: European Medicine Agency
FDA: Food and Drug Administration
GMP: Good Manufacturing Practice
hESC: Human Embryonic Stem Cell
hESC-I hNu: Human Neuron Induced From Human Embryonic Stem Cell
hESC-I hNuP: Human Neuronal Progenitor Induced From Human Embryonic Stem Cell
hNSC: Human Neural Stem Cell
ICM: Inner Cell Mass
iPS cell: Induced Pluripotent Stem Cell
MEF: Mouse Embryonic Fibroblast
miRNA: microRNA
NAM: Nicotinamide
RA: Retinoic Acid
SHH: Sonic Hedgehog
TH: Tyrosine Hydroxylase
VM: Ventral Mesencephelon
